# Supplementary material for: Resources used by young people to overcome mental distress in deprived settings in Latin America: a qualitative study
Source: BMC Psychol. 2025 Jul 4;13:727. doi: 10.1186/s40359-025-02830-w (PMC12231848; doi:10.1186/s40359-025-02830-w)
Supplement: Supplementary file 1 — Supplementary Material 1 [file 40359_2025_2830_MOESM1_ESM.docx]

**SUPPLEMENTARY MATERIALS**

**Supplementary material 1: Topic guide**

Questions:

I would like for you to think about moments over the last year, meaning, the past 12 months, in which you may have experienced emotional distress. With emotional distress we mean nervousness, anxiety, worry, or sadness, low mood, or lack of interest in your activities.

1. In these moments, what did you do to intentionally feel better?
   1. Why do you think it helped you? *(Explore for each resource mentioned)*
   2. How did (*answer to 1a*) help you to feel better? (*Explore for each resource mentioned*)
   3. Do you usually do this to feel better? Since when do you do this? Are there any specific situations when you do this? (*Explore for each resource mentioned*)
   4. *If the participant mentions youth groups/organisations*: Can you tell me more about this group/organisation? What is the name of the group/organisation?

2. Some people use other strategies to feel better. I will now go through a list of such activities/resources with you. When you feel emotionally distressed, what did you feel was helpful for you too in the last 12 months? (*Go through each resource on the list not mentioned in question 1*):

| Resources: |
| --- |
| Talking with family members (including via text, video calls) |
| Talking with friends (including via text, video calls) |
| Youth groups/organisations |
| Doing sports/exercise or physical activities |
| Doing artistic or creative activities |
| Using the internet |
| Using social media (Instagram, TikTok, Facebook, etc.) |
| Doing leisure activities (watching TV shows or movies, reading, listening to music) |
| Community services (helplines, university counselling services, social support organisations, etc.) |
| Talking with a psychologist, psychiatrist, or mental health professional |
| Ways of being (for example being positive, being empathic, being rational to make decisions, being flexible/relaxed, among other personal characteristics) |
| Learning and education activities (taking a course, learning a language) |
| Culinary activities (cooking, making desserts) |
| Spiritual and religious activities (praying) |
| Relaxation and resting activities (meditation, resting) |
| *(Add other resources mentioned by the participant below)* |
|  |
|  |
|  |
|  |

1. If the participant says “Yes” to a resource:

i. Why do you think it helped you?

ii. How did (*answer to 2ai*) help you to feel better?

iii. Do you usually do this intentionally to feel better? Since when do you do this and are there any specific situations when you do this? (*Explore for each resourced*)

iv. If the participant mentions youth groups/organisations: Can you tell me more about this group/organisation? What is the name of the group/organisation?

1. If the participant says “No” to a resource:
   - 1. Did you not do this, or did you do it and it did not help you?
2. Out of everything you have mentioned, during moments of more intense emotional distress, what has helped you the most to feel better?

3. Besides all the actions/activities/efforts that we just talked about and that helped you feel better, is there anything else that you wish you would have done when you were distressed and would have helped to make you feel better? For instance, other things that you wish you would have liked to but could not do because you did not have access or there were other barriers.

- 1. Did you try to access/ask for this help?

i. (*if they answer yes*) What barriers did you encounter?

ii. (*if they answer no*) Is there any reason you did not try?

4. When you filled in the questionnaires about 12 months ago, you reported being distressed, such as worrying or lack of interest in your activities, among others. (*Ask the following question according to the participant’s CRF results:*)

*Participant’s results in the CRF:*

*____ Recovered:*

However, in the last questionnaire you completed recently, you reported this distress has diminished or is gone. Why do you think you feel better now? (*if needed, follow-up asking about potential changes in living circumstances or life events*)

____ *Not recovered:*

And in the last questionnaire you completed recently, your answers suggest that there has not been much change. Why do you think there has been little or no change? (*if needed, follow-up asking about potential living circumstances or life events*)

- Is there anything else you would like to add about resources that help you to feel better?
- Do you have any additional questions about the project?

**Supplementary material 2: Codebook**

| **Category** | **Codes** | **Definition** | **Reported by** | |
| --- | --- | --- | --- | --- |
| Social resources | - Emotional support from relatives | Emotional support includes talking about their problems, asking for and receiving advice. Family includes core and extended family members but does not include a partner. | 57/112 |  |
|  | - Emotional support from friends and peers | Emotional support includes talking about their problems, asking for and receiving advice. Friends and peers include friends, coworkers, classmates, and people in the same age range. | 62/112 |  |
|  | - Emotional support from partner | Emotional support includes talking about their problems, asking for and receiving advice. Partner includes spouse, live-in partner, boyfriend/girlfriend. | 6/112 |  |
|  | - Emotional support from other close people or pets | Emotional support includes talking about their problems, asking for and receiving advice. Other close people include someone with an unspecified relationship with the participant (e.g. “someone close to me”), acquaintances (e.g. neighbours) or pets. | 6/112 |  |
|  | - Social support from relatives | Conducting activities with family members. Activities include talking, going out, doing activities together, using social media to communicate. It does not include receiving emotional support or talking about their problems. It does not include a partner | 24/112 |  |
|  | - Social support from friends and peers | Conducting activities with friends, coworkers, classmates, or peers. Activities include talking, going out, doing activities together, using social media to communicate. It does not include receiving emotional support or talking about their problems. | 49/112 |  |
|  | - Social support from partner | Conducting activities with a partner. Activities include talking, going out, doing activities together, using social media to communicate. It does not include receiving emotional support or talking about their problems. | 6/112 |  |
|  | - Social support from other close people | Conducting activities with someone with an unspecified relationship with the participant (e.g. “someone close to me”), acquaintances (e.g. neighbours) or pets. Activities include talking, going out, doing activities together, using social media to communicate. It does not include receiving emotional support or talking about their problems. | 7/112 |  |
|  | - Community groups | Being a member or participating in community or neighbourhood groups (e.g. church groups, fan clubs, sports clubs, etc.) | 15/112 |  |
|  | - Community help services | Receiving a help service (e.g. helplines, welfare, food banks) | 1/112 |  |
| Recreational and leisure activities | - Entertainment activities | Activities for leisure or hobbies such as reading, handcrafts, listening to music, playing video games, etc. It includes activities that use the internet but are not internet-based (e.g. streaming a movie, reading an eBook). | 73/112 |  |
|  | - Internet and social media | Use of the internet for leisure or entertainment. These activities require an internet connection to be used (e.g. social media, apps, blogs, etc.) | 54/112 |  |
|  | - Outdoors activities | Activities conducted outdoors for recreation purposes, such as going for a walk, strolling, etc. | 43/112 |  |
|  | - Travel | Travelling for recreation or entertainment. | 5/112 |  |
| Personal resources | - Personal strengthening | Traits or actions oriented to develop self-esteem, autonomy, self-efficacy, self-knowledge, etc. As well as maintaining attitudes and positive thoughts. | 61/112 |  |
|  | - Problem solving | Actions oriented to seek solutions to their problems, such as analysing the situation, thinking of potential solutions, organising their time and thoughts. It includes searching the internet or social media for solutions. | 31/112 |  |
|  | - Avoidance or distraction | Actively avoiding thinking or confronting the cause of the emotional distress. | 22/112 |  |
|  | - Selfcare | Activities related to order and cleanliness of objects and places. Also related to self-care activities (e.g. getting a haircut). | 3/112 |  |
|  | - Consuming food or beverages | Eating or drinking to deal with emotional distress. | 4/112 |  |
|  | - Substance use | Includes drugs, cigarettes, and alcohol. | 5/112 |  |
| Physical activity and sports | - Physical activity | All physical activity that is not a sport (e.g. running, walking, going to the gym, dancing, stretching, etc.) | 40/112 |  |
|  | - Individual sports | Sports that are not practised in teams (e.g. swimming, cycling, tennis, etc.) | 12/112 |  |
|  | - Group sports | Sports that are practised in groups (e.g. football, volleyball, basketball, etc.) | 17/112 |  |
| Relaxation activities | - Relaxation and self-regulation activities | Activities oriented to emotional expression, relaxation, and self-regulation (e.g. meditating, crying, taking a bath, receiving a massage, etc.) | 41/112 |  |
|  | - Resting | Activities oriented to resting (e.g. sleeping, taking a pause, etc.) | 31/112 |  |
| Arts activities | - Plastic arts | Activities such as sculpting, drawing, painting, knitting, graffiti, etc. | 21/112 |  |
|  | - Performance arts | Activities such as theatre, dance, circus, etc. | 6/112 |  |
|  | - Music | Activities such as playing instruments, songwriting, singing. It does not include listening to music. | 11/112 |  |
|  | - Literature | Activities such as writing prose, poetry, journaling, etc. It does not include reading or messaging. | 14/112 |  |
| Mental health services | - Psychologist or psychotherapist | Receiving mental health care from a psychologist or psychotherapist. | 29/112 |  |
|  | - Psychiatrist | Receiving mental health care from a psychiatrist. | 6/112 |  |
|  | - Counselling at educational institutions | Receiving mental health care or counselling from social workers, educators, counsellors, etc. | 6/112 |  |
| Spiritual activities | - | Activities related to religion or spirituality (e.g. praying, going to church, reading tarot) | 28/112 |  |
| Educational activities | - | Activities related to education (e.g. studying, taking courses, etc.) | 22/112 |  |
