# Supplementary material for: Resources used by young people to overcome mental distress in deprived settings in Latin America: a qualitative study
Source: BMC Psychol. 2025 Jul 4;13:727. doi: 10.1186/s40359-025-02830-w (PMC12231848; doi:10.1186/s40359-025-02830-w)
Supplement: Supplementary file 2 — Supplementary Material 2 [file 40359_2025_2830_MOESM2_ESM.docx]

**TITLE PAGE**

**Resources used by young people to overcome mental distress in deprived settings in Latin America: A qualitative study**

Mauricio Toyama^a^, Ana L. Vilela-Estrada^a^, Karen Ariza-Salazar^b^, Isabela Osorio Jaramillo^b^, Daniela Ramirez-Meneses^a^, Sumiko Flores^a^, Adriana Carbonel^a^, Natividad Olivar^c^, Fernando Luis Carbonetti^c^, Catherine Fung^d^, Diliniya Stanislaus Sureshkumar^d^, Luis Ignacio Brusco^c^, Carlos Gómez-Restrepo^b,e,f^, Francisco Diez-Canseco^a^, Stefan Priebe^g^

Affiliations

^a^ CRONICAS Centre of Excellence in Chronic Diseases, Universidad Peruana Cayetano Heredia, Lima, Peru

^b^ Department of Clinical Epidemiology and Biostatistics, Pontificia Universidad Javeriana, Bogota, Colombia

^c^ Department of Psychiatry and Mental Health, School of Medicine, University of Buenos Aires, Buenos Aires, Argentina

^d^ Unit for Social and Community Psychiatry, Wolfson Institute of Population Health, Queen Mary University of London, London, United Kingdom

^e^ Department of Psychiatry and Mental Health, Pontificia Universidad Javeriana, Bogota, Colombia

^f^ Hospital Universitario San Ignacio, Bogotá, Colombia

^g^ Centre for Psychosocial Medicine, University of Hamburg, Germany

Corresponding author: Diliniya Stanislaus Sureshkumar (dilistani9@gmail.com)

**ABSTRACT**

**Background:** Adolescence and young adulthood are critical stages for developing mental health problems. However, the ability to cope with adversity can help them manage emotional distress and overcome mental health conditions. This qualitative study aims to describe the resources reported by urban Latin-American young people as useful to overcome mental distress.

**Methods:** We conducted semi-structured interviews with a purposive sample of 112 adolescents (age 15-16 years) and young adults (age 20-24 years) who participated in a cohort study in deprived urban areas of Bogotá, Buenos Aires and Lima. All of them had symptoms of depression (PHQ-8>9) and/or anxiety (GAD-7>9) at their inclusion in the cohort. The interviews took place after a 12-month follow-up.

**Results:** To overcome mental distress, participants reported most social and personal resources, as well as recreational and leisure activities, followed by physical activities and sports, and relaxation activities. All resources were mentioned by both genders and age groups. However, several resources were reported more frequently by female and young adult participants than by male and adolescents. Social and personal resources, physical activity and sports, recreational and leisure activities, and educational activities were reported more frequently by participants who recovered from their symptoms of depression and/or anxiety after 12 months. Conversely, arts activities were reported more by participants who did not recover from their symptoms.

**Conclusions:** The results show that young people in deprived urban settings in Latin America use a range of resources and activities they consider helpful to overcome mental distress. Policies for improving mental health of young people should consider promoting the most helpful activities and resources. Further research may explore how exactly these resources help to reduce mental distress and how they can be strengthened.

**KEYWORDS:** qualitative research, resilience, coping strategies, youth, Latin America, depression, anxiety.

**BACKGROUND**

The United Nations defines young people as the age group between 15 and 24 years [1]. In recent years, there has been a decline in young people’s mental health. A study with data from 46 countries found that, prior to the COVID-19 pandemic, wellbeing among adolescents showed a global decline from 2015 to 2018 [2]. During the pandemic in Latin America and the Caribbean (LAC), 46% of young people aged 13 to 29 reported reduced motivation to engage in once pleasurable activities and expressed pessimism about the future (46% for women and 31% for men) [3]. Moreover, in LAC, there has been a marked increase in the prevalence of mental health disorders, with rates rising to 19% among adolescents aged 15 to 19 and 20% among young adults aged 20 to 24, between 2019 and 2021 [4].

Adolescence and young adulthood are vulnerable developmental stages for mental health. Adolescents experience biological, social, and psychological changes that make them vulnerable to developing mental health disorders [5, 6]. Firstly, the brain section involved in response inhibition, emotional regulation, and decision-making is not fully matured, which increases their engagement in risky behaviour, exposes them to stressful life events, and lowers their ability to cope with these events [5-8]. Secondly, young adults are in a stage of instability since there are recurrent and often involuntary changes in their work, social, and love lives [9]. Identity, occupational trajectory, and lifetime goals are still being defined during this period, which can bring confusion, uncertainty, and hopelessness [9]. Therefore, both adolescents and young adults are more prone to experiencing mental health problems [10].

Despite developmental changes and stressful life events, adolescents and young adults can achieve good outcomes. Resilience theories suggest that having enough protective factors can help moderate or reduce the risk of an adverse outcome and promote positive ones [11]. Resilience is defined as “harnessing the resources needed to sustain well-being in the face of stress or adversity” [12]. These resources act as protective factors and can be individual, such as coping skills and self-efficacy; social resources, such as community support; or existential, such as finding purpose during or after adversity [11, 12].

Adolescents and young adults with resilience resources have a lower risk of depression and anxiety [12, 13]. Coping strategies like problem-focused coping, positive reframing, optimism, humour, and support-seeking are linked with a lower risk of mental health problems in young people [13-15]. Conversely, avoidant coping strategies (e.g., substance abuse and other withdrawal/distraction activities that keep the individual from directly addressing the stressful event) and emotional coping strategies have been associated with an increased risk of mental health problems [13, 15-17].

A qualitative study involving young Spanish people with affective disorders revealed various strategies for dealing with emotional distress, including resources that reinforce individuality, such as isolating oneself or looking for solutions to their problems by themselves; seeking support on social networks; and engaging in activities promoting physical activity and self-care, such as sports and creative endeavours, respectively [18]. Similarly, research on Latin American youth reports that resources that address individual aspects like personal development, emotional expression, distraction, and skill-building, and social factors, such as being in a group and social support, assist young adults in managing emotional discomfort [19, 20]. Interestingly, activities providing distraction, group interactions, and a sense of being understood were the most frequently reported by young people, such as personal and social resources and physical and sports activities, suggesting their significance in coping with emotional distress [20].

Research has been conducted on resources to help young people overcome emotional distress in populations experiencing academic pressure [21], health conditions [12], and affective disorders in Europe [18]. In these studies, young people reported using strategies such as seeking the support of family and friends, participating in spiritual activities, and engaging in dynamics that make it possible to externalise discomfort, introspection, and optimism to cope with emotional distress. However, based on the available information, there are very few studies exploring this topic among youth populations that have experienced depression and anxiety in Latin America. Furthermore, there are even less studies focused on youth populations from deprived areas, where they are more exposed to risk factors for mental distress [22].

This paper aims to describe the resources reported by young people, from vulnerable environments in three Latin American cities participating in the OLA research programme, as useful in their own experience to overcome moments of mental distress. Additional aims are to describe the most salient differences in the reported resources based on the participants' age group, gender, city, and recovery status of the participants after the 12-month follow-up of the cohort.

**METHODS**

**Study design**

The study was embedded within the research programme ‘Building resilience and resources to reduce depression and anxiety in young people from urban neighbourhoods in Latin America (OLA)’ [23], a collaboration between Queen Mary University of London (UK), Universidad de Buenos Aires (Argentina), Pontificia Universidad Javeriana in Bogota (Colombia) and Universidad Peruana Cayetano Heredia in Lima (Peru). The overall aim of the programme is to identify which characteristics, resources and activities help young people living in urban environments in Latin America to prevent or recover from depression and/or anxiety.

This qualitative study uses a qualitative descriptive design, allowing to give a voice to participants, providing insight into their experiences and perceptions [24]. Data collection was conducted primarily through individual semi-structured interviews. We aimed to conduct a minimum of thirty interviews per country, ninety in total, as suggested in the literature to ensure richness of data [25].

**Setting**

The study was conducted in Buenos Aires, Bogota and Lima, the capital cities of Argentina, Colombia, and Peru, respectively. Similar to the overarching OLA programme, this study focused on adolescents and young adults from deprived urban areas in the three cities, defined through national indicators. The details on how participants were included in the OLA programme have been published elsewhere [23].

**Participants**

A sample of participants was selected from the cohort of adolescents and young adults included in the OLA programme. The inclusion criteria for the cohort were: being 15-16 or 20-24 years of age, having the capacity to provide assent/consent, living in an eligible area (within the 50% poorest areas defined by the programme), having symptoms of depression and/or anxiety during the baseline assessment of the cohort, defined as having a score > 9 in the Patient Health Questionnaire-8 (PHQ-8) [26] and General Anxiety Disorder-7 (GAD-7) [27], respectively.

Additional inclusion criteria for this study were providing informed consent to participate in the interviews and, for adolescents, receiving informed consent from a parent/legal guardian. In addition, interviewed participants completed the 12-month follow-up of the cohort. The selection of participants followed a purposive sampling approach and aimed to have a balance of gender, age group and recovery from symptoms of depression and/or anxiety at the 12-month time-point mark in each city.

**Data collection tools**

Demographic data was collected as part of the cohort's baseline questionnaire. The research team created a topic guide for the interviews. The guide explored which resources the interviewees used when facing emotional distress over the past year. The explored resources (e.g. arts activities, sports and physical activities) were based on the results of the qualitative study conducted in the formative phase of the OLA programme [20]. The structure of the topic guide followed a freelisting approach, followed by an exploration of specific categories of resources not mentioned by the participants in their initial response, to assess if they did not use these resources or they had forgotten to mention them.

The topic guide was piloted with seven young people (3 in Bogota, 2 in Lima, and 2 in Buenos Aires) of similar characteristics as the participants the OLA programme. Their comments and suggestions were used to improve the questions. The same topic guide was used in all three countries (see Supplementary Material 1).

**Procedures**

Participants who accepted to take part in the interviews during the cohort recruitment and completed the 12-month follow-up were contacted by the research team and invited to participate. If the participant confirmed their interest in being interviewed, a virtual or in-person meeting was set up, according to their availability. The interviews were audio recorded and transcribed.

Data collection was conducted between August 2022 and November 2023 by nine members of the research team (ALVE, IOJ, DRM, SF, NO, FLC, LHP, SCL, and FE) (4 in Buenos Aires, one in Bogotá, and four in Lima). Interviews were conducted remotely through different platforms (e.g. Zoom, Google Meets, Microsoft Teams). The interview duration had an average of 38 min (SD = 22 min).

**Data analysis**

Audio recordings were transcribed verbatim and analysed through a deductive content analysis [28] using NVivo 14 software (Lumivero, LLC). The data analysis was conducted in parallel to the data collection. In an initial phase, 7 members of the research team (AVL, KAS, IOJ, LHP, SCL, FE, and MCR) (2 in Buenos Aires, 3 in Bogotá and 2 in Lima) familiarised themselves with a small group of transcriptions (2 per country) and created an initial set of codes. The process to develop the codes followed a similar procedure as a previous qualitative study conducted as part of the OLA programme [20] Afterwards, the researchers met to discuss and agree on a standard codebook for the analysis (see Supplementary Material 2). The same seven researchers conducted the coding of the remaining interviews, plus 4 more (DRM, SF, FF, LF) (2 in Buenos Aires and two in Lima). The teams of each country coded their own interviews, meeting regularly to discuss the process, solve any potential doubts, and add new codes they considered necessary. Finally, five researchers from Lima (MT, ALV, DRM, SF, AC) created summaries for each code, including information from all three countries.

**RESULTS**

**Participants’ characteristics**

In total, 112 participants were interviewed: forty-two in Buenos Aires, forty in Bogotá and thirty in Lima. Overall, we achieved a balance of recovered (50.9%) and non-recovered participants (49.1%). Regarding age group and gender, slightly over half of the participants were young adults (57.1%) and female (54.5%). The sociodemographic characteristics are found in Table 1.

*Table 1. Participants’ sociodemographic characteristics*

|  | **Buenos Aires**  (n = 42) | **Bogotá**  (n = 40) | **Lima**  (n = 30) | **Total**  (n = 112) |
| --- | --- | --- | --- | --- |
| **Age group** | | | | |
| Adolescents | 12 (29%) | 21 (53%) | 15 (50%) | 48 (42.9%) |
| Young adults | 30 (71%) | 19 (47%) | 15 (50%) | 64 (57.1%) |
| **Gender** | | | | |
| Male | 16 (38%) | 20 (50%) | 14 (47%) | 50 (44.6%) |
| Female | 26 (62%) | 20 (50%) | 15 (50%) | 61 (54.5%) |
| Non-binary | 0 | 0 | 1 (3%) | 1 (0.9%) |
| **Recovery status** | | | | |
| Recovered* | 24 (57%) | 19 (47%) | 14 (47%) | 57 (50.9%) |
| Not Recovered** | 18 (43%) | 21 (53%) | 16 (53%) | 55 (49.1%) |
| *Recovered is defined as having a score < 10 in both scales at the 12-month follow-up.  **Not recovered is defined as having a score > 9 in the PHQ-8 and/or GAD-7 at the 12-month follow-up. | | | | |

**Reported resources**

The reported resources found useful by participants when facing emotional distress in the last year were grouped into nine categories (see Table 2). The most reported were social resources, recreational and leisure activities, and personal resources, reported, each of them, by over three-quarters of interviewed participants. This is followed by physical activity and sports, and relaxation activities, reported by around half of the participants. Other resources include arts activities, mental health services, and spiritual and educational activities, reported by a third or less of participants. A detailed definition of the categories and sub-categories of resources can be found in Supplementary Material 2.

*Table 2. Categorisation of resources reported by participants*

| **Category of resources** | **Included resources** | **Reported by** |
| --- | --- | --- |
| Social resources | - Emotional support (from relatives, friends and peers, partner, or other) - Social support (from relatives, friends and peers, partner, or other) - Community groups - Community help services | 105/112 |
| Recreational and leisure activities | - Entertainment activities - Internet and social media - Outdoors activities - Travel | 95/112 |
| Personal resources | - Personal strengthening - Problem-solving - Avoidance or distraction - Selfcare - Consuming food or beverages - Substance use | 87/112 |
| Physical activity and sports | - Physical activity - Individual sports - Group sports | 68/112 |
| Relaxation activities | - Relaxation and self-regulation activities - Resting | 66/112 |
| Arts activities | - Plastic arts - Performance arts - Music - Literature | 38/112 |
| Mental health services | - Psychologist or psychotherapist - Psychiatrist - Counselling at educational institutions | 37/112 |
| Spiritual activities | - | 28/112 |
| Educational activities | - | 22/112 |
|  |  |  |

**Social resources**

Most participants reported using social resources when experiencing emotional distress. This resource was reported slightly more by young adults, females and recovered participants. In addition, participants from Buenos Aires and Bogotá reported using it more than participants from Lima.

The most reported social resource was receiving emotional support. The participants reported receiving support from friends or peers, and relatives, usually mothers or siblings. The emotional support was described as conversations where the interviewees could talk about their problems, receive advice or vent. Regarding the benefits of this resource, they mentioned feeling supported and not judged.

*“I always tell my mom everything that is going on, if I fail a test, everything, always. She’s like my personal psychologist (…) She’s always like ‘good, I can tell you studied, but if it didn’t go well, prepare for the supplementary test’ She’s always like that, she never judges me because something didn’t go well, she’s always supporting me in everything” (Participant 41, young adult, male, recovered, Buenos Aires)*

Another reported resource was receiving social support and was more commonly associated with friends or peers than with relatives. This type of support consisted of spending time with them, going out or conducting different activities without discussing personal matters.

*“Sometimes I would go out with my friends, my family, with them I had moments of peace, tranquillity, joy, I would laugh a lot and say, ‘everything is okay, it will get better’ and it helped a lot, having nice moments with my family and friends” (Participant 109, young adult, female, recovered, Lima)*

A few participants also mentioned belonging to community groups as a social resource. These groups were more commonly religious or artistic, and some were political groups or groups with shared interests, such as video games or music.

*“(The theatre group) is a space in which I was offered a lot of support and normally they offer support because curiously the people who go there usually have some problems or seek to vent” (Participant 53, adolescent, female, not recovered, Bogotá)*

The least reported social resources were receiving emotional and social support from other people or pets. In addition, only one participant mentioned using a community service, specifically a helpline for domestic violence.

**Recreational and leisure activities**

The second most frequently reported resource comprised recreational and leisure activities, reported by most participants. This resource was reported almost equally across gender, age group, recovery status, and city.

Recreational activities are divided into four types: entertainment activities, internet and social media use, outdoor activities, and travel. Entertainment activities were the most reported, being mentioned by almost two-thirds of participants. Within this group of activities, the most common were listening to music, cooking, and watching movies, series, or anime. Cooking was reported more by participants in Lima, and watching movies, series, or anime was mentioned more by participants in Bogotá and Lima. Other mentioned activities included reading, going to the movies, and playing video games.

*“When I feel very distressed, I make desserts, all kinds of desserts, even last year, around September, I tried to sell empanadas, so I improved my recipe, and when I felt distressed or low, like I didn’t want to do anything, I went into the kitchen to cook things like desserts” (Participant 105, young adult, female, not recovered, Lima).*

Internet and social media use for recreational purposes was reported slightly more by females, young adults, and non-recovered participants. The most reported activities were watching videos, more commonly mentioned by participants in Buenos Aires and Lima, and checking social media, more mentioned by participants in Bogotá. Another mentioned activity was browsing the internet.

*“(Social media) entertains me and makes me stop thinking about some things, problems” (Participant 5, adolescent, female, not recovered, Buenos Aires)*

Over one-third of the participants reported outdoor activities as a useful resource when facing emotional distress. It was reported in similarly by participants from the three cities and in terms of gender and age group, but slightly more common among recovered participants. The most reported activity was going out to a public space, such as a park. Other activities mentioned were cycling or skating, perceived as recreational by the interviewees.

*“I was feeling restless and felt like I had to make decisions right away, without thinking, but with all that noise inside [my head], I go for a walk, I walk and while walking I think about stuff, and I feel like it goes away and everything falls into place” (Participant 81, young adult, male, recovered, Bogotá)*

Finally, a small group of participants from Bogotá and Lima reported travelling as a resource.

**Personal resources**

The third most frequently reported resource were personal resources, mentioned by over three-quarters of the participants. These resources were more commonly mentioned by young adults and females and slightly more by recovered participants.

Within these resources, the most reported were personal strengthening, problem-solving and avoidance. Personal strengthening was reported by over half of the participants and consisted of having a positive attitude when facing emotional distress, implementing previously useful strategies to alleviate the distress, such as thinking situations happen for a reason, validating their own emotions, talking to themselves, and reflecting.

*“I have always been a positive person, I may feel distressed and all, but I try to always look at the positive, otherwise I would sink in myself, so I need to be positive and say ‘okay, I feel bad, but tomorrow this is going to happen, the day after I have to do this’ and it is something that thrills me, sort of like a plan, basically” (Participant 53, adolescent, female, not recovered, Bogotá)*

Problem-solving was reported by slightly over a third of the participants, and the most common strategy was seeking information to solve or understand the situation causing emotional distress. The sources of information mentioned were the internet, videos, and groups on social media. Other strategies mentioned were reorganising their routine to alleviate the distress and analysing the situation to identify solutions.

Some participants reported avoidance or distraction as a resource, through other activities that help take their minds off the situation causing emotional distress. A few participants also mentioned ignoring or not thinking about the situation as a strategy to deal with distress.

*“(When I’m experiencing distress) I distract myself thinking about something else, I could be listing names by initials following the alphabet, I would think about that, or other things, like counting from 1 to 10” (Participant 86, adolescent, female, recovered, Lima)*

Finally, very few participants also reported some personal resources such as self-care, eating or drinking, as well as substance use when experiencing emotional distress.

**Physical activity and sports**

Engaging in physical activity when experiencing emotional distress was reported by less than two thirds of interviewed participants. This resource was reported more by young adults and participants from Buenos Aires and Bogotá than Lima. In addition, it was mentioned in similar proportions by males and females and slightly more by recovered participants.

Physical activity was reported by over a third of the participants, specifically going to the gym and going for a walk or run as the most mentioned resources. This is followed by group sports, where football and basketball were the most mentioned. Lastly, individual sports were also mentioned by some participants, including cycling, tennis, swimming, skating and pole dancing.

*“Basically group (sports), because there I have conversation with friends about the topic that was causing distress, or just to clear my head” (Participant 33, young adult, male, recovered, Buenos Aires)*

**Relaxation activities**

Relaxation and self-regulation activities were reported by slightly over half of the interviewed participants. Female, young adult, and recovered participants mentioned these activities more frequently. The most common activities included sleeping or resting, meditation and breathing exercises.

*“What I do when I feel bad or feel sad or want to repress it or be alone, I try to sleep; when I feel like that or tired of a situation, I try to sleep, lay on the bed, not get up, because it makes me feel better” (Participant 111, young adult, female, recovered, Lima)*

**Arts activities**

Arts activities were mentioned as a resource by a third of the participants. These activities were more frequently used by female and unrecovered participants, and in similar proportions across age groups and cities. Among the activities reported, the most mentioned were plastic arts, specifically drawing and painting. Other less commonly mentioned were writing, music (e.g. playing an instrument or singing), and performing arts (e.g. dancing, theatre). Plastic and performing arts, as well as writing were more reported by participants from Bogotá, while music was more reported by participants from Buenos Aires.

*“Writing when I feel bad, because I heard that when you write, you let go of what you have inside, and drawing, I like everything about drawing, to make it perfect, and in the end, all the concentration to reach that goal” (Participant 70, adolescent, female, recovered, Bogotá)*

**Mental health services**

The use of mental health services was mentioned by a third of the participants, mostly women and young adults. This resource was reported in similar proportions by recovered and unrecovered participants and was more commonly mentioned by interviewees from Buenos Aires and Lima, than Bogotá.

The most common type of mental health professional participants reported seeing was a psychologist or psychotherapist. In addition, a few participants also reported receiving care from a psychiatrist. Finally, a few interviewees reported receiving counselling in their educational institutions (e.g., university, school, institute).

**Spiritual activities**

Spiritual activities were reported by a quarter of the participants, more frequently mentioned by women and young adults, as well as participants from Lima. It was reported in similar proportions by recovered and unrecovered participants. Among the activities reported, almost all participants mentioned praying. A few participants also reported “talking to God” or a higher being.

*“I don’t have a trusting relationship with my family to tell them how I feel, so talking to God makes me feel heard” (Participant 86, adolescent, female, recovered, Lima)*

**Educational activities**

Finally, educational activities were the least mentioned resource when facing emotional distress. Women, young adults, and recovered participants reported this resource more frequently. In addition, participants from Buenos Aires reported it more.

Among the educational activities mentioned, the most frequent was taking a course, more commonly a language or a course about a personal interest (e.g. photography, marketing). In addition, some participants mentioned focusing on academic activities when facing emotional distress, such as studying and doing homework.

*“I’ve done online marketing courses, and those type of things, it helps me to be distracted” (Participant 24, young adult, female, not recovered, Buenos Aires)*

**DISCUSSION**

This study identifies what resources adolescents and young adults find useful when facing emotional distress. The resources reported were varied and usually combining different resources at a time. Overall, the most reported were social and personal resources, as well as recreational and leisure activities, followed by physical activities and sports, and relaxation activities. Regarding differences in the use of resources, all resources explored were mentioned by both genders and age groups. However, several resources were reported more frequently by female and young adult participants (i.e. social and personal resources, relaxation, spiritual and educational activities, and mental health services), and no resource was reported more by males nor adolescents than their counterparts.

In addition, all the categories of resources were identified across all cities, with minor differences in some resources being more common than others (e.g. music was more reported in Buenos Aires, spirituality in Lima, and performing arts in Bogotá). This result reveals the taxonomy of resources generated from this study can probably be generalised to similar settings across Latin America, and other deprived settings.

In terms of recovery status, some resources were reported more frequently by participants who recovered from their symptoms of depression and/or anxiety after 12 months of being included in the cohort. These resources include social and personal resources, physical activity and sports, recreational and leisure activities, and educational activities. In addition, only arts activities were reported more by participants who did not recover from their symptoms. Our discussion will focus on these resources.

The emotional and social support provided by family and friends is a very important and used resource for the interviewees, which aligns with the literature on the importance of social support in different populations [12, 16, 18, 21, 29]. However, it is important to note the interviewed participants highlighted not only the emotional support received, but also the support while engaging in recreational activities with people. This illustrates how people engage with different types of resources, deployed either in isolation or combined to act as protective factors to promote their wellbeing [11].

Regarding personal resources, having a positive attitude and problem-solving were the most common. These types of resources have been described before as active coping strategies which help directly address the events causing distress [15, 30], and have been found to decrease the risk of depression and suicidal ideation [13]. In contrast, avoidance or distraction, another common resource reported, has been described as maladaptive in the literature, as it is an emotion-focused coping strategy that has been correlated with an increased risk of depression [13, 30].

Another important resource mentioned were physical activities and sports, which have been documented in different reviews to have a positive impact on young people’s mental health, protecting against anxiety and depression, and promoting self-esteem and confidence [31-35]. This result has also been found in previous qualitative studies with young people experiencing emotional distress [18, 20].

Relaxation activities, such as breathing exercises and meditation, have been documented as effective in reducing depression and anxiety symptoms in young people [36, 37], while sleep interventions have shown a small but significant effect in improving anxiety and depression in young adults [38]. Meanwhile, educational activities may provide skills and purpose, which can function as protective factors for depression and anxiety [12].

Conversely, arts activities as a resource were reported more by participants who did not recover from their symptoms. This result highlights the importance of delving deeper into the mechanisms in which arts activities may promote wellbeing in this population. Some reviews conclude arts interventions may have promising results for young people to alleviate their emotional distress [39, 40]. However, some may ruminate or criticise themselves while engaging in some arts activities, which can lead to further distress [30, 41]. Therefore, further research is required to understand which aspects of arts activities may be more effective in promoting wellbeing than others.

**Implications**

Overall, our findings have several implications. Firstly, they provide an overview of the more and less frequently used resources reported by young people with depression and/or anxiety symptoms living in lower-income urban settings in Latin America. The range of resources is varied and may be deployed depending on individual preferences or the context. Therefore, there is no standard that applies to all young people. However, some of the most reported resources could inform future interventions and programmes to promote wellbeing in this population. For instance, focusing on strengthening social and personal resources, which were some of the most reported resources.

Secondly, the participants' experiences reveal how resources are usually used in combination (e.g. social support and recreational activities, relaxation activities and personal resources), which is an important aspect to take into consideration when studying resilience and resources. National and local governments should create policies and programmes to provide opportunities and spaces for young people to strength and use these resources. For instance, promoting sports organisations may provide a space for young people to use physical activity as a resource, but also to strengthen their social support system, and use other personal resources.

Thirdly, further studies are required to understand the association between the use of these resources and the reduction of anxiety and/or depression symptoms. Finally, mental health services are among the resources that young people report as less used. This may be associated with barriers to access these services, stigma regarding mental health, among other factors. Therefore, it is important to conduct additional studies to understand better why young people do not use mental health services and the barriers they face in accessing mental health care.

**Strengths and limitations**

This study builds on previous efforts to explore and characterise the resources used by young people to overcome mental distress [20], by focusing specifically on people with symptoms of depression and/or anxiety measured by commonly used screening tools. This allowed a richer analysis of the interviews by incorporating additional levels, such as the cities and the recovery status. Another strength of the study is the use of a consistent methodology across all countries, which allows to collect a rich set of qualitative data to analyse.

However, this study also has limitations. The sample of participants does not necessarily reflect or represent the experiences of all young people living in low-income settings in Latin America. Their living conditions and exposure to stressors may vary from person to person and influence the resources used and reported. Nevertheless, due to the qualitative design of the study, the focus was on exploring their experiences in-depth, instead of achieving representation. Moreover, the data collected provides an overview of the resources used as reported by the participants, but we cannot assess if its use was effective in lowering their emotional distress.

In addition, due to technical problems with the software used, an inter-rater reliability score could not be calculated. Lastly, it is important to take into consideration social desirability when reviewing the results. The interview setting may have influenced and diminished the report of some resources socially perceived as maladaptive, such as substance use.

**CONCLUSIONS**

The results of our study describe the resources used by young people with depression and/or anxiety symptoms from low-income urban settings in Latin America. Social and personal resources, as well as recreational and leisure activities, were the most frequently reported. For participants who recovered from their symptoms, the reported resources align with previous evidence about protective factors for depression and anxiety, including social and personal resources, physical activity and sports, recreational and leisure activities, and educational activities. Conversely, arts activities were more commonly reported by participants who did not recover from their symptoms. Further studies analysing the association between these resources and depression and anxiety symptoms may allow a better understanding of how these protective factors work according to the young people’s characteristics.

**LIST OF ABBREVIATIONS**

OLA programme Building resilience and resources to reduce depression and anxiety in young people from urban neighbourhoods in Latin America

PHQ-8 Patient Health Questionnaire-8

GAD-7 General Anxiety Disorder-7

**DECLARATIONS**

**Ethics approval and consent to participate**

The study was conducted in accordance with the Declaration of Helsinki. The protocol and all procedures were approved by the Institutional Review Boards (IRB) of Universidad de Buenos Aires on October 2nd, 2020, Pontificia Universidad Javeriana on November 20th, 2020 (FM-CIE-1138-20), and Universidad Peruana Cayetano Heredia on November 16th, 2020 (Constancia 581-33-20), as well as the Research Ethics Committee of Queen Mary, University of London, on November 16th, 2020 (QMERC2020/02).

All participants provided written informed consent before taking part in the study.

**Consent for publication**

Not applicable.

**Availability of data and materials**

The datasets used and/or analysed during the current study are available from the corresponding author on reasonable request.

**Competing interests**

The authors declare that they have no competing interests.

**Funding**

This work was supported by the Medical Research Council (grant number: MR/S03580X/1).

**Authors' contributions**

SP, LIB, CGR and FDC devised the project, the main conceptual ideas and design of the study. CF and DSS overviewed the project execution. ALVE, IOJ, DRM, SF, NO and FLC conducted the data collection. MT, ALVE, KAS, IOJ, DRM, SF, AC, NO and FLC conducted the data analysis. MT wrote the manuscript with input from all authors. All authors reviewed and approved the final manuscript.

**Acknowledgements**

The authors would like to acknowledge Liliana Hidalgo-Padilla, Santiago Cesar Lucchetti, Fernando Esnal, Maria Camila Roldan, Florencia Flores, and Lucia Flores for their contributions to the paper.

**REFERENCES**

1. United Nations. Youth. [cited 2024 08 20]; Available from: <https://www.un.org/en/global-issues/youth>.

2. Marquez J, Long E. A Global Decline in Adolescents' Subjective Well-Being: a Comparative Study Exploring Patterns of Change in the Life Satisfaction of 15-Year-Old Students in 46 Countries. *Child Indic Res* 2021;24:1251-92.

3. UNICEF. El impacto del COVID-19 en la salud mental de adolescentes y jóvenes. [cited 2024 08 20]; Available from: <https://www.unicef.org/lac/el-impacto-del-covid-19-en-la-salud-mental-de-adolescentes-y-j%C3%B3venes>.

4. Institute for Health Metrics and Evaluation. GBD Compare. Mental disorders. Both sexes, 15-19 years. 2024 [updated 2024; cited 2024 08 20]; Available from: <http://ihmeuw.org/6ij0>.

5. Sigelman CK, Rider EA. *Life-span: Human Development (9th ed.)*. Boston: CENGAGE Learning; 2018.

6. Steinberg L. Cognitive and affective development in adolescence. *Trends Cogn Sci* 2005;9(2):69-74.

7. Blakemore SJ. Development of the social brain during adolescence. *Q J Exp Psychol (Hove)* 2008;61(1):40-9.

8. Casey B, Caudle K. The Teenage Brain: Self Control. *Curr Dir Psychol Sci* 2013;22(2):82-7.

9. Arnett JJ, Zukauskiene R, Sugimura K. The new life stage of emerging adulthood at ages 18-29 years: implications for mental health. *Lancet Psychiatry* 2014;1(7):569-76.

10. Anderson K, Priebe S. Concepts of Resilience in Adolescent Mental Health Research. *J Adolesc Health* 2021;69(5):689-95.

11. Fergus S, Zimmerman MA. Adolescent resilience: a framework for understanding healthy development in the face of risk. *Annu Rev Public Health* 2005;26:399-419.

12. Salsman JM, Rosenberg AR. Fostering resilience in adolescence and young adulthood: Considerations for evidence-based, patient-centered oncology care. *Cancer* 2024;130(7):1031-40.

13. Dai Q, Smith GD. Resilience to depression: Implication for psychological vaccination. *Front Psychiatry* 2023;14:1071859.

14. Pritchard ME, Wilson GS, Yamnitz B. What predicts adjustment among college students? A longitudinal panel study. *J Am Coll Health* 2007;56(1):15-21.

15. Salih S, Mahmoud SS, Abudeyah MA, Albeladi FI, Mohsen WM, Hayyan AI, et al. Stressors and coping strategies among medical students in Jazan, Saudi Arabia: A cross-sectional study. *J Family Med Prim Care* 2023;12(9):2075-81.

16. Fullerton DJ, Zhang LM, Kleitman S. An integrative process model of resilience in an academic context: Resilience resources, coping strategies, and positive adaptation. *PLoS One* 2021;16(2):e0246000.

17. Smith MM, Saklofske DH, Keefer KV, Tremblay PF. Coping Strategies and Psychological Outcomes: The Moderating Effects of Personal Resiliency. *J Psychol* 2016;150(3):318-32.

18. Martorell-Poveda MA, Martinez-Hernaez A, Carceller-Maicas N, Correa-Urquiza M. Self-care strategies for emotional distress among young adults in Catalonia: a qualitative study. *Int J Ment Health Syst* 2015;9:9.

19. Gomez-Restrepo C, Casasbuenas NG, Ortiz-Hernandez N, Bird VJ, Acosta MPJ, Restrepo JMU, et al. Role of the arts in the life and mental health of young people that participate in artistic organizations in Colombia: a qualitative study. *BMC Psychiatry* 2022;22(1):757.

20. Toyama M, Godoy-Casasbuenas N, Olivar N, Brusco LI, Carbonetti F, Diez-Canseco F, et al. Identifying resources used by young people to overcome mental distress in three Latin American cities: a qualitative study. *BMJ Open* 2022;12(8):e060340.

21. Heinen I, Bullinger M, Kocalevent RD. Perceived stress in first year medical students - associations with personal resources and emotional distress. *BMC Med Educ* 2017;17(1):4.

22. Navarra-Loli J, Moscoso M, G C-DlC. La investigación de la depresión en adolescentes en el Perú: una revisión sistemática. *Liberabit* 2017;23(1):57-74.

23. Priebe S, Fung C, Brusco LI, Carbonetti F, Gomez-Restrepo C, Uribe M, et al. Which resources help young people to prevent and overcome mental distress in deprived urban areas in Latin America? A protocol for a prospective cohort study. *BMJ Open* 2021;11(9):e052339.

24. Doyle L, McCabe C, Keogh B, Brady A, McCann M. An overview of the qualitative descriptive design within nursing research. *J Res Nurs* 2020;25(5):443-55.

25. Morse JM. Determining Sample Size. *Qualitative Health Research* 2000;10(1):3-5.

26. Shin C, Lee SH, Han KM, Yoon HK, Han C. Comparison of the Usefulness of the PHQ-8 and PHQ-9 for Screening for Major Depressive Disorder: Analysis of Psychiatric Outpatient Data. *Psychiatry Investig* 2019;16(4):300-5.

27. Spitzer RL, Kroenke K, Williams JB, Lowe B. A brief measure for assessing generalized anxiety disorder: the GAD-7. *Arch Intern Med* 2006;166(10):1092-7.

28. Bengtsson M. How to plan and perform a qualitative study using content analysis. *NursingPlus Open* 2016;2:8-14.

29. Ayed N, Toner S, Priebe S. Conceptualizing resilience in adult mental health literature: A systematic review and narrative synthesis. *Psychol Psychother* 2019;92(3):299-341.

30. Alosaimi FD, Almufleh A, Kazim S, Aladwani B. Stress-coping strategies among medical residents in Saudi Arabia: A cross-sectional national study. *Pak J Med Sci* 2015;31(3):504-9.

31. Eime RM, Young JA, Harvey JT, Charity MJ, Payne WR. A systematic review of the psychological and social benefits of participation in sport for children and adolescents: informing development of a conceptual model of health through sport. *Int J Behav Nutr Phys Act* 2013;10:98.

32. English M, Wallace L, Evans J, Diamond S, Caperchione CM. The impact of sport and physical activity programs on the mental health and social and emotional wellbeing of young Aboriginal and Torres Strait Islander Australians: A systematic review. *Prev Med Rep* 2022;25:101676.

33. Miller K, Morley C, Fraser BJ, Gall SL, Cleland V. Types of leisure-time physical activity participation in childhood and adolescence, and physical activity behaviours and health outcomes in adulthood: a systematic review. *BMC Public Health* 2024;24(1):1789.

34. Panza MJ, Graupensperger S, Agans JP, Dore I, Vella SA, Evans MB. Adolescent Sport Participation and Symptoms of Anxiety and Depression: A Systematic Review and Meta-Analysis. *J Sport Exerc Psychol* 2020;42(3):201-18.

35. Pascoe MC, Bailey AP, Craike M, Carter T, Patten R, Stepto NK, et al. Exercise interventions for mental disorders in young people: a scoping review. *BMJ Open Sport Exerc Med* 2020;6(1):e000678.

36. Gonzalez-Valero G, Zurita-Ortega F, Ubago-Jimenez JL, Puertas-Molero P. Use of Meditation and Cognitive Behavioral Therapies for the Treatment of Stress, Depression and Anxiety in Students. A Systematic Review and Meta-Analysis. *Int J Environ Res Public Health* 2019;16(22).

37. Reangsing C, Punsuwun S, Schneider JK. Effects of mindfulness interventions on depressive symptoms in adolescents: A meta-analysis. *Int J Nurs Stud* 2021;115:103848.

38. Chandler L, Patel C, Lovecka L, Gardani M, Walasek L, Ellis J, et al. Improving university students' mental health using multi-component and single-component sleep interventions: A systematic review and meta-analysis. *Sleep Med* 2022;100:354-63.

39. Buechner H, Toparlak SM, Ostinelli EG, Shokraneh F, Nicholls-Mindlin J, Cipriani A, et al. Community interventions for anxiety and depression in adults and young people: A systematic review. *Aust N Z J Psychiatry* 2023;57(9):1223-42.

40. Keiller E, Tjasink M, Bourne J, Ougrin D, Carr CE, Lau JYF. A systematic review of dramatherapy interventions used to alleviate emotional distress and support the well-being of children and young people aged 8-18 years old. *JCPP Adv* 2023;3(3):e12145.

41. Bell IH, Marx W, Nguyen K, Grace S, Gleeson J, Alvarez-Jimenez M. The effect of psychological treatment on repetitive negative thinking in youth depression and anxiety: a meta-analysis and meta-regression. *Psychol Med* 2023;53(1):6-16.
